# Genomic analysis of exceptional responders to radiotherapy reveals somatic mutations in *ATM*

**DOI:** 10.18632/oncotarget.14400

**Published:** 2016-12-31

**Authors:** Jennifer Ma, Jeremy Setton, Luc Morris, Pedro Blecua Carrillo Albornoz, Christopher Barker, Benjamin H. Lok, Eric Sherman, Nora Katabi, Kathryn Beal, Ian Ganly, Simon N. Powell, Nancy Lee, Timothy A. Chan, Nadeem Riaz

**Affiliations:** ^1^ Department of Radiation Oncology, Memorial Sloan Kettering Cancer Center, New York, NY, USA; ^2^ Department of Surgery, Memorial Sloan Kettering Cancer Center, New York, NY, USA; ^3^ Department of Medical Oncology, Memorial Sloan Kettering Cancer Center, New York, NY, USA; ^4^ Department of Pathology, Memorial Sloan Kettering Cancer Center, New York, NY, USA; ^5^ Human Oncology and Pathogenesis, Memorial Sloan Kettering Cancer Center, New York, NY, USA

**Keywords:** somatic ATM mutations, radiation therapy

## Abstract

Radiation therapy is a mainstay of cancer treatment, yet the molecular determinants of clinical response are poorly understood. We identified exceptional responders to radiotherapy based on clinical response, and investigated the associated tumor sequencing data in order to identify additional patients with similar mutations. Among head and neck squamous cell cancer patients receiving palliative radiotherapy at our institution, we identified one patient with documented complete metabolic response. Targeted sequencing analysis of the tumor identified a somatic frame-shift mutation in *ATM*, a gene known to be associated with radio-sensitivity in the germline. To validate the association of somatic *ATM* mutation with radiotherapy response, we identified eight patients with *ATM* truncating mutations who received radiotherapy, all of whom demonstrated excellent responses with a median local control period of 4.62 years. Analysis of 22 DNA repair genes in The Cancer Genome Atlas (TCGA) data revealed mutations in 15.9% of 9064 tumors across 24 cancer types, with *ATM* mutations being the most prevalent. This is the first study to suggest that exceptional responses to radiotherapy may be determined by mutations in DNA repair genes. Sequencing of DNA repair genes merits attention in larger cohorts and may have significant implications for the personalization of radiotherapy.

## INTRODUCTION

Patients with metastatic head and neck cancer or locally advanced disease and poor performance status often receive palliative radiotherapy (RT). Palliative RT often alleviates symptoms but rarely provides long-term loco-regional control or prolonged survival [1, 2]. Although full-dose RT may provide for more durable loco-regional control, the increased acute toxicity associated with standard treatment typically outweighs the benefits in patients with limited life expectancies. Occasionally, some patients respond better than anticipated to palliative RT and achieve long-term disease control. The mechanistic basis for these exceptional responses to palliative RT remains poorly understood.

**Figure 1:**
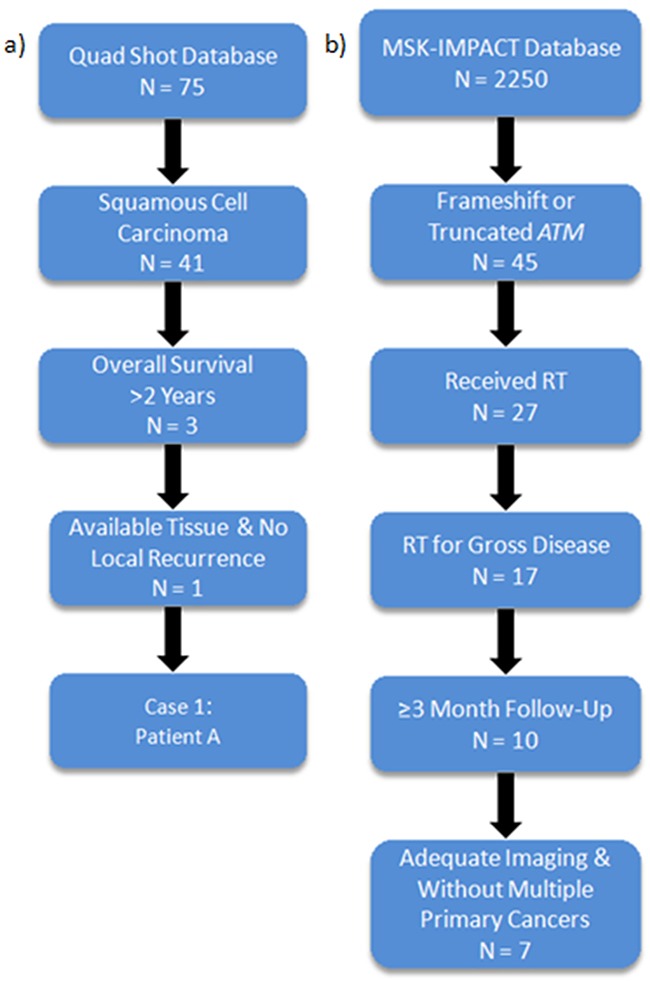
Identification of long-term survivors with *ATM* frameshift or truncating mutations demonstrating good responses to radiotherapy **a**. Institutional database of palliative Quad Shot RT revealed 1 SCC patient with long-term disease control. **b**. Institutional sequencing database (MSK-IMPACT) identifies 8 patients with good responses to RT.

Although there are several germline syndromes that predispose to marked radiation sensitivity, to our knowledge, alterations in genes associated with these syndromes have not been linked to tumor hypersensitivity to RT [3]. Here we examine patients at our institution who received palliative RT for head and neck squamous cell cancers (HNSCC) and identified a patient with long-term disease control (Figure 1a). Targeted sequencing analysis of the tumor revealed a frame-shift mutation in *ATM*, a gene centrally involved in the DNA damage response. To further investigate the role of *ATM* in tumor response, we then examined a separate institutional database of patients who underwent targeted sequencing analysis and identified eight patients with similar *ATM* mutations that received palliative RT, all of whom appeared to have excellent responses (Figure 1b).

## RESULTS

Here we briefly describe the relevant clinical histories of three of the eight to demonstrate the exceptional responses to palliative radiotherapy observed. We also discuss genomic analysis of tumor sequencing data to identify patients with a DNA repair mutation that may be a determinant of clinical response.

### Case 1: Patient A (HNSCC with long-term complete metabolic response)

**Figure 2:**
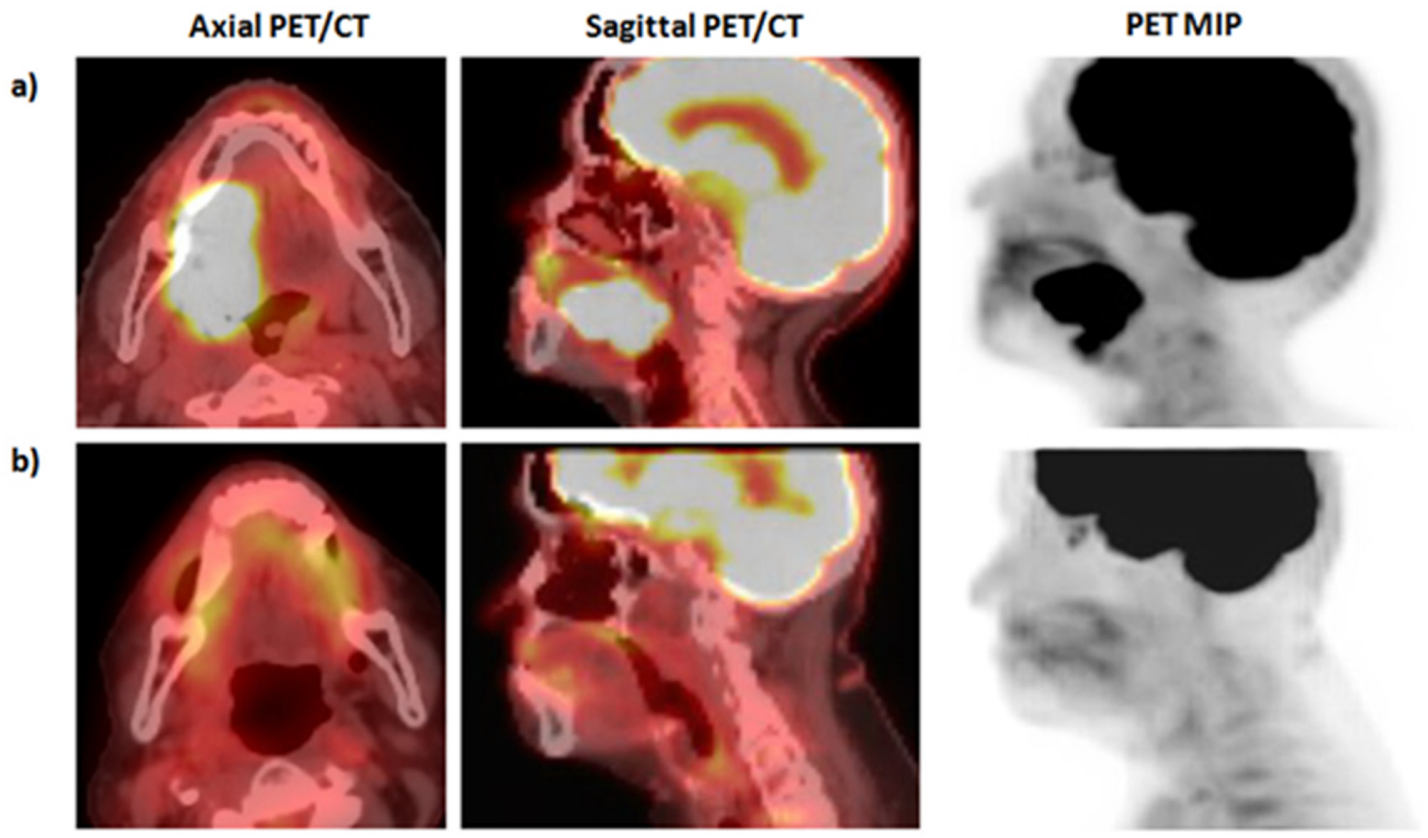
Pre- and post-therapy imaging for patient A *MIP: Fludeoxyglucose (^18^F) positron emission tomography (FDG-PET) maximum intensity projection (MIP). a) Pre-radiotherapy PET/CT demonstrates primary oral tongue SCC with SUV of 35.4 with FDG avid level Ib and II lymph nodes. b) Three month post-RT PET/CT demonstrates complete resolution of FDG avid malignancy.

Patient A is a 96 year old female with a p16 positive, moderately differentiated, invasive SCC of the right lateral oral tongue. Right-sided level I and II lymph nodes (SUV 2.3) were also present and were suspicious for metastatic disease (Figure 2a). Patient A was treated with cetuximab 500 mg/m^2^ for 7 months until disease progression. Patient A was then treated with 44.4 Gy of RT in 12 fractions with the “Quad Shot” regimen, with complete metabolic resolution of disease on a PET/CT performed 3 months post-RT (Figure 2b). A CT scan 8 months after RT and accompanying physical exam demonstrated no evidence of recurrence. She was last seen in clinic 34 months after RT with no evidence of recurrence and she has not needed additional therapy since that time.

Targeted sequencing [4] using a next generation sequencing panel of cancer genes demonstrated that patient A had a frameshift mutation in the *ATM* gene at position 1455, along with 29 other somatic mutations. Of note, the tumor is also p53 wild-type with a mutation in RAD50 (R519H) and a frameshift mutation in MLH1 at position 64.

### Institutional tumor sequencing database

Due to the strong evidence linking *ATM* to radiation sensitivity in the constitutional setting [5, 6], we searched our institutional MSK-IMPACT (Memorial Sloan Kettering-Integrated Mutation Profiling of Actionable Cancer Targets) database of 2,250 patients who underwent targeted panel sequencing to identify other patients with truncating mutations in *ATM*. Forty-five other patients, harboring a variety of primary malignancies, were found to have frame-shift or truncating *ATM* mutations. Of this cohort, seventeen patients underwent radiotherapy to grossly evident tumor. Ten of these patients had a minimum of 3 months of follow-up, of which 2 cases were excluded, one due to inadequate imaging and another due to multiple synchronous primary malignancies. The remaining eight patients demonstrated long-term disease control within the RT treatment field (Table 1). We review one of the above 8 cases with long-term loco-regional control from radiotherapy and briefly discuss another two of the remaining seven cases below.

**Table 1:** Characteristics of 8 Patients with *ATM* mutations identified from both the Quad Shot and MSK-IMPACT databases. LC: local control

Patient	Disease	*ATM* Mutation	RT Target	RT Dose / Fraction(Gy)	In-Field Recurrence	Duration of LC(Months)
A	Head and Neck Squamous Cell Cancer	I1453^1^	Oral Tongue	44.4 / 12	No	34.5
B	Endometrial Cancer	G1370^¥^	L3-L4	36 / 12	No	52.5
			Right Presacral	36 / 12	No	33.7
C	Non-Small Cell Lung Cancer	Q71^1^	Whole Brain	37.5 / 15	Yes	43.2
D	Breast Cancer	D1548M^1^	Whole Brain	37.5 / 15	Yes	41.0
E	Colon Cancer	R1875*	Abdominal Metastasis	30 / 10	No	3.8
F	Colon Cancer	R1898*	Pelvis	37.5 / 15	No	3.9
G	Non-Small Cell Lung Cancer	I1441^1^Q1331H	Neck & Upper Mediastinum	50.4 / 28	No	3.1
H	Thyroid Cancer	L2738*	Thyroid & Upper Mediastinum	70 / 33	No	5.16

°Note: Patient B had two separate sites treated at two different time points; *: nonsense mutation, ^1^: frameshift mutation, ^¥^: splice site mutation

### Case 2: Patient B (Endometrial Cancer with long-term response)

Patient B is a 68 year old woman diagnosed with FIGO stage IB, Grade 2 endometrial cancer who underwent a total abdominal hysterectomy, bilateral salpingo-oophorectomy, and pelvic lymph node sampling. She recurred 2 years later and underwent gross total surgical resection.

**Figure 3:**
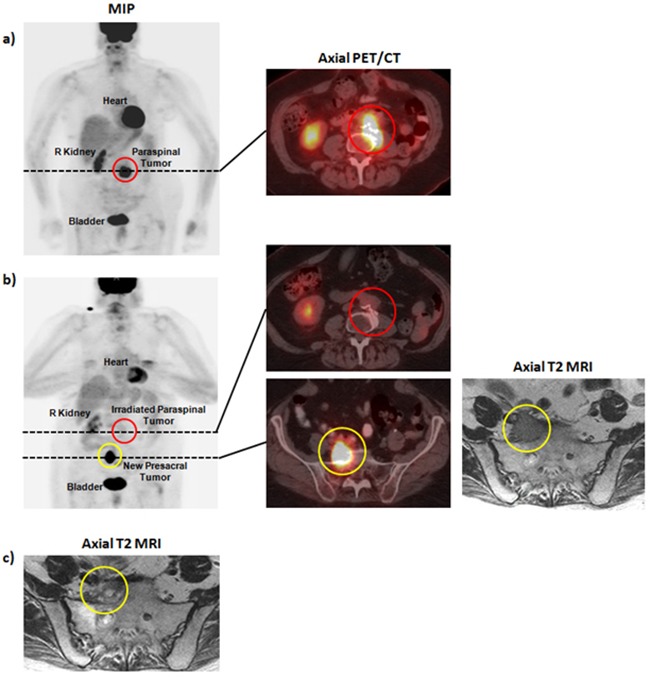
Pre- and post-therapy imaging for patient B **a**. Pre-radiotherapy PET/CT demonstrating a para-spinal mass involving the L4 vertebral body. **b**. Post-radiotherapy PET/CT and MRI demonstrating treatment response at L3-L4 and an out-of-field recurrence in a right pre-sacral lymph node. **d**. Post-radiotherapy MRI demonstrating reduction in size of the right pre-sacral lymph node.

Re-staging studies 3 years after initial diagnosis demonstrated a left para-spinal recurrence involving the L4 vertebral body (Figure 3a). She received palliative RT to 36 Gy in 12 fractions. Subsequent MR imaging demonstrated stable disease and a PET/CT scan demonstrated complete metabolic response to treatment. She was noted to have an out-of-field recurrence in a pre-sacral lymph node 1 year later (Figure 3b). She received 36Gy in 12 fractions to this second site of disease. Subsequent imaging demonstrated reduction in size of this lesion without evidence of recurrence (Figure 3c). She is currently without evidence of in-field recurrence of the L3-L4 lesion for over 4.3 years, and demonstrates stable disease of the right pre-sacral lesion for 2.8 years.

### Case 3: Patient C (Lung Cancer with long-term response)

Patient C is a 64 year old woman diagnosed with EGFR mutated NSCLC. MR imaging of the brain demonstrated a 5 mm enhancing brain metastasis in the right temporal lobe. She received whole-brain radiotherapy (WBRT) to 37.5 Gy, and was subsequently enrolled on a clinical trial with a pan-HER inhibitor. She developed progression of disease in her pleura 3 years later. An MRI of the brain performed over 3.5 years after diagnosis unfortunately demonstrated a new left frontal lobe metastasis, documenting an in-field recurrence. She demonstrated local control for 43 months with an overall survival of 48 months. A graded prognostic assessment was utilized to predict expected survival outcomes for patients with brain metastasis based on age, KPS, presence of extracranial metastasis, and number of brain lesions. The assessment predicted an overall survival of 6.5 months for Patient C, as compared to the observed 48 months.

### Genomic analysis of NHEJ pathway mutations in TCGA

**Figure 4:**
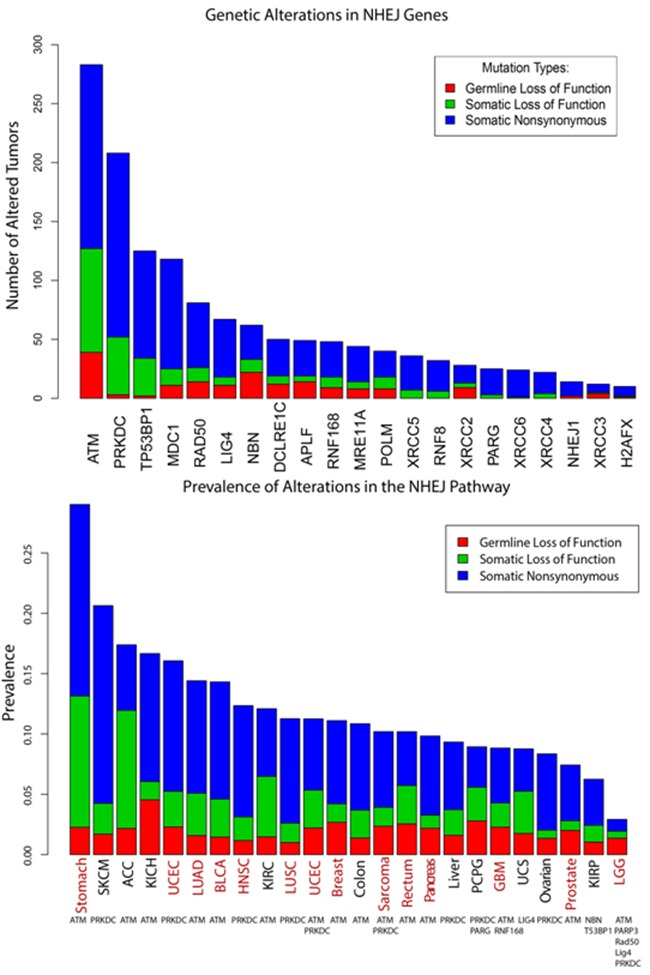
Analysis of TCGA data to identify frequency of DNA repair mutations **a**. Analysis of 22 DNA repair related genes involved in NHEJ. a) 9064 tumor samples were examined across 24 cancer types, with 15.9% of tumors exhibiting at least one alteration among the 22 genes. *ATM* was the most highly mutated, with 19.6% of tumors exhibiting some type of *ATM* alteration. **b**. Prevalence of NHEJ pathway alterations are listed by cancer type. The most commonly mutated gene within the pathway, for each cancer type, is listed below the corresponding cancer type. *ATM* was the most commonly mutated gene in fifteen of the 24 cancer types (62.5%). Red text identifies cancers for which radiation is used as standard of care as either definitive or adjuvant treatment. Adrenocortical carcinoma [ACC], Bladder Urothelial Carcinoma [BLCA], Glioblastoma multiforme [GBM], Head and Neck squamous cell carcinoma [HNSC], Kidney Chromophobe [KICH], Kidney renal clear cell carcinoma [KIRC], Kidney renal papillary cell carcinoma [KIRP], Brain Lower Grade Glioma [LGG], Lung adenocarcinoma [LUAD], Lung squamous cell carcinoma [LUSC], Pheochromocytoma and Paraganglioma [PCPG], Skin Cutaneous Melanoma [SKCM], Uterine Corpus Endometrial Carcinoma [UCEC], Uterine Carcinosarcoma [UCS].

We next examined The Cancer Genome Atlas (TCGA) data to identify the frequency of mutations in 22 DNA repair genes involved in the non-homologous end joining (NHEJ) pathway [8] across 24 cancer types (Table 2). Genes in NHEJ, similar to *ATM*, are strongly linked to *in vitro* sensitivity to IR across multiple cell types. Of 9,064 tumor samples, 15.9% exhibited at least one genetic alteration (germline loss of function mutation, somatic loss of function mutation, or somatic missense mutation) in one of the 22 NHEJ genes (Figure 4a) [9, 10]. Of these, *ATM* was the most highly mutated gene, with 19.6% of tumors with an NHEJ pathway alteration exhibiting some type of *ATM* mutation. Additionally, we examined the prevalence of NHEJ pathway alterations by cancer type and identified the most commonly mutated gene within the pathway, for each cancer type. Of 24 cancer types with NHEJ mutations, 15 (62.5%) cancer types revealed ATM to be the most commonly mutated gene (Figure 4b, Supplemental Figure 1).

**Table 2:** Twenty-two genes involved in the NHEJ pathway and their associated functions

Gene(HUGO)	Function
APLF	Chromatin-binding checkpoint protein [36]
ATM	DSB signaling [37]
DCLRE1C	End-processing [38]
LIG4	Main DSB ligating enzyme [39]
MDC1	DSB signaling; recruits DNA damage response elements [40]
MRE11A	DSB signaling of MRN complex [41]
NBN	DSB signaling of MRN complex [42]
NHEJ1	Ligase accessory factor [43]
PARG	Catabolism of PAR [44]
POLM	Polymerase; gap-filling [45]
PRKDC	DSB signaling; DNA-dependent protein kinase catalytic subunit [46]
RAD50	DSB signaling; MRN complex [47]
RNF168	DSB signaling; 63-linked histone poly-ubiquitination of H2AX; (downstream RNF88, upstream BRCA1) [48]
RNF8	Ubiquinates H2AX [49]
TP53BP1	DSB signaling [50]
XRCC2	DNA break and crosslink repair [51]
XRCC3	DNA break and crosslink repair [51]
XRCC4	Ligase accessory factor [52]
XRCC5	Binds to DSB end/Ku heterodimer [53]
XRCC6	Binds to DSB end/Ku heterodimer [54]
H2AFX	DSB signaling [55]

## DISCUSSION

Ionizing radiation (IR) induces a number of DNA aberrations including base damage, single-strand breaks, and double-strand breaks (DSB) [11]. DSBs are thought to be the most lethal of these lesions, with each Gray of IR producing between 20-40 DSBs. ATM, a Ser/Thr kinase, serves as a key signaling hub in the DNA DSB damage response [12, 13]. ATM exists in the cell as an inactive homo-dimer which is activated by genotoxic stress, at which point the dimer dissociates and an ATM monomer is recruited to the site of the DSB by the Mre11-Rad50-NBS1 (MRN) complex [12, 14]. The activated ATM monomer phosphorylates H2AX and several other proteins in order to facilitate the recruitment of DSB repair machinery.

A wealth of evidence accumulated over the past 40 years indicates that ATM plays a critical role in the response to IR in experimental systems. First, cell lines derived from patients with *ataxia-telangiectasia* syndrome or carriers for the gene are known to exhibit marked sensitivity to IR [5, 6]. Knockdown of *ATM* is often used as a control in experiments to determine if other genes increase sensitivity to radiation [15]. Pharmacologic inhibition of *ATM in vitro* with several different selective inhibitors also leads to marked radio-sensitization and is ineffective in *ATM* deficient cell lines [16–18]. In an orthotopic xenograft model of glioblastoma multiforme, intra-tumoral injections of an ATM inhibitor led to significantly increased survival when combined with RT, although this effect appeared superior in p53-mutated cells [19].

A recent phase II trial of patients with metastatic prostate cancer demonstrated increased sensitivity of patients with *ATM* aberrations to a poly(adenosine diphosphate [ADP]-ribose) polymerase (PARP) inhibitor [20]. Although the germline ataxia-telangiectasia syndrome is due to compound heterozygosity, haplo-insufficiency in *ATM* is known to increase radio-sensitivity as well as increase risk of cancer development [6, 21, 22]. Interestingly, patients with mono-allelic *ATM* aberrations affecting the C-terminal phosphoinositide 3-kinase (PI3K) catalytic domain were identified in the aforementioned phase II study as sensitive to another class of DNA damaging agents, namely PARP inhibitors, suggesting a clinically relevant haplo-insufficient phenotype.

Rare germline genetic syndromes involving DSB repair genes have been associated with exquisite radio-sensitivity and severe adverse reactions to radiation therapy [3]. In addition to *ATM*, germline mutations in *MRE11* has been shown to be predictive of radiotherapy response in bladder cancer [23, 24], while inherited defects in DSB repair genes NBS1 and Lig4 have been linked to fatal complications from low doses of radiotherapy [25–27]. High *MRE11* expression levels on immunohistochemistry have been associated with improved cancer-specific survival in radiotherapy patients compared to high *MRE11* expression in patients undergoing cystectomy alone, also suggesting a role for epigenetic alterations in *MRE11* in radiotherapy response [23]. These cases suggest that common toxicities from radiotherapy may be heritable and has prompted a search for common and rare variants of inherited defects associated with toxicity [28]. However, tumor response to somatic genetic alterations in DNA repair genes have not been adequately explored. To our knowledge, this is the first report of multiple clinical cases with alterations in *ATM* demonstrating dramatic clinical responses to palliative radiotherapy.

*ATM* is one of only 22 genes recurrently mutated in 3 or more cancers (renal cell, lung adenocarcinoma, and prostate cancer) [8]. Furthermore, truncating mutations are present in at least 1% of tumors in over 12 different cancers. As more patients undergo sequencing as part of their workup, with some undergoing sequencing of multiple genes, identifying patients with alterations in DNA repair genes may offer novel opportunities for RT in previously palliative settings. These cases highlight the possibility that functional alterations in key DNA DSB repair genes may result in increased sensitivity to radiotherapy.

**Figure 5:**
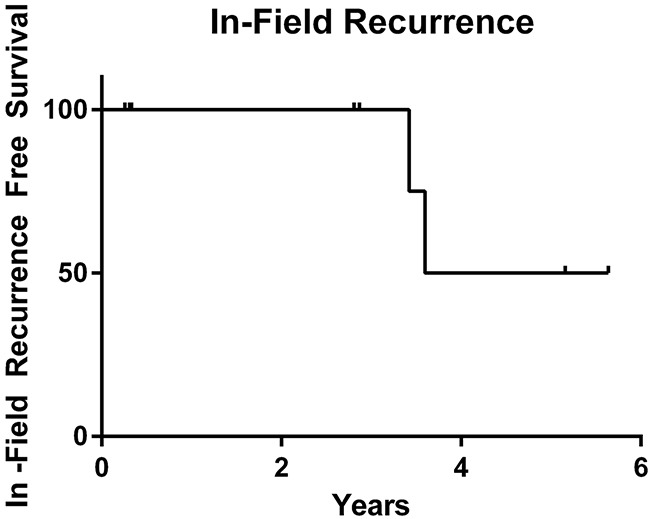
Kaplan-Meier analysis of local recurrence data for 8 patients with truncating and frameshift *ATM* mutations treated with radiotherapy to gross disease, in years Median time to in-field local recurrence is 4.62 years (range: 0.26-5.64). Two of the eight patients developed an in-field recurrence.

Here we have identified an unusual long-term response of an oral cavity SCC to palliative RT, with an associated frameshift *ATM* mutation. Patient A had a complete metabolic response to radiotherapy with biopsy confirmed absence of disease 1 year post-RT. Although p16 status is associated with an improved prognosis in oropharynx cancers, it is not associated with outcomes in squamous cell cancers of the oral cavity [29]. More importantly, p16 status in cancers outside the oropharynx does not appear to be linked to HPV infection [30]. We subsequently identified 8 patients with truncating mutations in *ATM* who received radiotherapy to gross disease and had tumor sequencing performed. The local control and overall survival rates in our cohort of patients with *ATM* mutations appear to be superior to historic controls, although these types of comparisons are limited. Median time to local recurrence for this population was 4.62 years, with two of the eight patients developing local recurrence within the radiation field (Figure 5). Patient C was treated with WBRT for a NSCLC brain metastasis and demonstrated disease control for more than 3.5 years. The other 6 patients included in this study exhibited similar exceptional responses to radiotherapy.

These cases highlight the possibility of utilizing sequencing information to personalize radiotherapy treatment decisions in patients with genetic aberrations in DNA repair genes. The data presented here, along with evidence for mutations in DNA repair genes driving response to other DNA damaging agents, strongly supports further exploration of mutations in these genes in larger cohorts. Ultimately, data from this case series will need to be validated in a larger cohort of patients to definitively define the role of mutations in DNA repair genes in radiotherapy response. If more definitive evidence emerges suggesting radio-sensitivity in these patients, previously abandoned uses of large field, low-dose radiotherapy such as whole abdominal treatment or liver treatment could be reconsidered.

## MATERIALS AND METHODS

The retrospective review of outcomes with corresponding genetic alterations was approved by the institutional review board (IRB).

### Palliative radiotherapy for HNSCC

Our institutional preference for palliative radiotherapy to the head and neck is based on the RTOG 8502 regimen, which consists of two fractions of 3.7Gy per day for two days, and colloquially named the “Quad Shot.” The Quad Shot is typically repeated 3 to 4 times, with one cycle every 2-4 weeks [31, 32]. A database of patients who received palliative Quad Shot RT to the head and neck at our institution between 02/2005 and 06/2014 was used to identify patients who demonstrated long-term disease control [33].

Of the 75 patients who received palliative Quad Shot RT, 40 had squamous cell carcinoma (SCC) (Figure 1a). We focused on SCC and excluded other histologies such as thyroid and salivary carcinoma as they can have prolonged clinical courses. Of the 40 SCC patients, 6 patients had an overall survival of greater than 1 year, and 2 patients had an overall survival of greater than 2 years. Of the latter group, one patient had a cutaneous SCC and developed loco-regional recurrence shortly after RT. The remaining long-term survivor is discussed above.

### Targeted sequencing

The MSK-IMPACT is an institutional effort to genotype patients for the determination of effective targeted therapies. It provides tumor genomic mutation profiling with a custom hybridization capture-based NGS assay [4]. The MSK-IMPACT targeted sequencing assay was performed on DNA from formalin-fixed, paraffin-embedded tumor samples with patient-matched normal blood samples. Bar-coded libraries from the tumor and normal blood samples were captured, sequenced, and subjected to a custom pipeline in order to identify somatic mutations. Deep sequencing of all exons and a custom 341 cancer-associated gene panel of selected introns was performed. An updated panel contained an expanded list of 410 genes. All exons had a minimum depth of coverage of 100X. Somatic mutations were identified via automated comparison of alterations from tumor and matched normal samples. Mutational load was calculated via determination of the number of somatic non-silent protein-coding mutations, excluding structural rearrangements and copy number gene alterations. The mutational burden was calculated based on the 341 gene panel for all patients, including those for whom the newer 410 gene panel was applied [34].

### Analysis of TCGA data

Somatic mutation and copy number data for 24 cancer types analyzed by the TCGA was obtained from Broad Institute’s GDAC Firehose (http://gdac.broadinstitute.org). Two authors manually curated a list of DNA repair genes to identify genes that are associated with NHEJ (N.R., S.N.P) [8]. Germline events were obtained from a collaborator. Data analysis was performed in the R statistical environment version 3.2.4 using custom scripts.

### Prevalence of altered tumors in the NHEJ pathway

We extracted from the TCGA Portal (https://gdc-portal.nci.nih.gov) all bam files that were used for WXS (Whole Exome Sequencing) in 24 cancer types, for a total of 9064 tumors. We then made a customized R script to calculate the number of tumors altered in the NHEJ pathway (using a list of 22 genes) per cancer type, and divided the alterations in three groups: germ line loss of function, somatic loss of function and somatic non-synonymous. The germ line alterations were extracted from a curated database (Ruomu et. Al., “Pan-cancer sequencing analysis reveals frequent germline mutations in cancer genes”, (2015), unpublished) [35] while the somatic alteration MAF files were extracted from the TCGA firehose (http://gdac.broadinstitute.org) for each cancer. For each cancer type, and for each alteration class, the number of corresponding altered tumors was divided by the total number of bam files assigned to that particular cancer type that were used for WXS. The resulting percentage, germ line loss of function, somatic loss of function, or somatic non-synonymous alterations is referred to as prevalence.

For each cancer type, we identified the most altered gene by counting the number of altered tumors per gene, across all three alteration types.

### Statistics

Local recurrence was calculated from date of RT to date of death or last known follow-up. The Kaplan-Meier method was used to determine time to loco-regional recurrence within the radiation field.

## SUPPLEMENTARY FIGURE


