# Supplementary material for: Genomic analysis of exceptional responders to radiotherapy reveals somatic mutations in ATM
Source: Oncotarget. 2016 Dec 31;8(6):10312–23. doi: 10.18632/oncotarget.14400 (PMC5354661; doi:10.18632/oncotarget.14400)
Supplement: Supplementary file 1 [file oncotarget-08-10312-s001.pdf]

Genomic analysis of exceptional responders to radiotherapy reveals somatic mutations in *ATM*

SUPPLEMENTARY FIGURE

	Germline Loss of Function	Somatic Loss of Function	Somatic Non-synonomous
ACC	0.032608696	0.097826087	0.054347826
BLCA	0.016990291	0.031553398	0.099514563
BRCA	0.028735632	0.016283525	0.070881226
CESC	0.029508197	0.029508197	0.108196721
COAD	0.018475751	0.025404157	0.071593533
GBM	0.027777778	0.02020202	0.045454545
HNSC	0.015686275	0.019607843	0.094117647
KICH	0.045454545	0.015151515	0.106060606
KIRC	0.026548673	0.050147493	0.056047198
KIRP	0.013888889	0.013888889	0.041666667
LGG	0.01754386	0.005847953	0.009746589
LIHC	0.018666667	0.021333333	0.056
LUAD	0.015817223	0.035149385	0.096660808
LUSC	0.016096579	0.016096579	0.086519115
OV	0.018058691	0.006772009	0.065462754
PAAD	0.032786885	0.010928962	0.06557377
PCPG	0.027932961	0.027932961	0.033519553
PRAD	0.022088353	0.008032129	0.046184739
SARC	0.023529412	0.015686275	0.062745098
SKCM	0.017021277	0.025531915	0.163829787
STAD	0.022675737	0.111111111	0.16553288
UCEC	0.029520295	0.031365314	0.060885609
UCS	0.01754386	0.035087719	0.035087719
READ	0.025477707	0.031847134	0.044585987

Supplementary Figure 1: TCGA data listing prevalence of NHEJ pathway alterations among 24 cancer types. Data are graphed in Figure 4.
